# Identify and Validate the Transcriptomic, Functional Network, and Predictive Validity of FBXL19-AS1 in Hepatocellular Carcinoma

**DOI:** 10.3389/fonc.2020.609601

**Published:** 2020-12-03

**Authors:** Dingdong He, Xiaokang Zhang, Xinyu Zhu, Narayani Maharjan, Yingchao Wang, Ping Luo, Chunzi Liang, Jiancheng Tu

**Affiliations:** Department & Program of Clinical Laboratory Medicine, Center for Gene Diagnosis, Zhongnan Hospital of Wuhan University, Wuhan, China

**Keywords:** hepatocellular carcinoma, immune infiltrates, pathogenesis, biomarker, FBXL19-AS1

## Abstract

Hepatocellular carcinoma (HCC) is one of the most common neoplastic diseases worldwide. Available biomarkers are not sensitive enough for the diagnosis of HCC, hence seeking new biomarkers of HCC is urgent and challenging. The purpose of this study was to investigate the role of F-box and leucine-rich repeat protein 19-antisense RNA 1 (FBXL19-AS1) through a functional network and inquire into its diagnostic and prognostic value in HCC. A comprehensive strategy of genomic data mining, bioinformatics and experimental validation was used to evaluate the clinical value of FBXL19-AS1 in the diagnosis and prognosis of HCC and to identify the pathways in which FBXL19-AS1 might be involved. FBXL19-AS1 was up-regulated in HCC tissues, and its high expression was associated with TNM stage and poor prognosis of HCC patients. The combination of FBXL19-AS1 and alpha-fetoprotein (AFP) in plasma could prominently improve the diagnostic validity for HCC. FBXL19-AS1 might stabilize FBXL19 to reduce the amount of macrophage M1, and then promote the occurrence and development of HCC. Meanwhile, FBXL19-AS1 might participate in regulating HCC related pathways through FBXL19-AS1-miRNA-mRNA network. Our findings indicated that FBXL19-AS1 not only serves as a potential biomarker for HCC diagnosis and prognosis, but also might be functionally carcinogenic.

## Introduction

Liver cancer is a common malignant cancer globally. According to the latest global cancer statistics, liver cancer ranks sixth and fourth in morbidity and mortality among all types of cancers, respectively (1). Hepatocellular carcinoma (HCC) accounts for 70%–85% of primary liver cancers and is the most common form of liver cancer. Recent years have witnessed a great gain in treatment methods of HCC, such as surgical resection, liver transplantation, adjuvant therapy, and interventional therapy (2, 3). The 5-year survival rate of HCC patients with early diagnosis and appropriate treatment or intervention is more than 50% (4), but for those who are diagnosed and treated after the relevant symptoms appear, the 5-year survival rate is only 14% (5). Currently, although alpha-fetoprotein (AFP) has been reported to be a valid marker for the clinical diagnosis and prognosis of HCC, its value remains unsatisfactory in early diagnosis. To improve the outcome and prognosis of HCC patients, it is essential to find more effective biomarkers to improve the early diagnosis of HCC (6).

Studies have found that non-coding RNAs (ncRNAs), such as microRNAs (miRNAs) and long non-coding RNAs (lncRNAs), are involved in regulating proliferation, invasion and metastasis of HCC cells, providing a novel perspective for the diagnosis and prognosis of HCC (7). LncRNAs could not only function as competitive endogenous RNA (ceRNA) to suppress the activity of miRNAs, but also as inducible endogenous RNA to facilitate the functions of miRNAs by protecting miRNAs from degradation (8, 9). In addition, the antisense lncRNA could be paired with its sense mRNA through partial base complementation, affecting the expression and functions of the mRNA.

In the present study, The Cancer Genome Atlas liver hepatocellular carcinoma (TCGA LIHC) dataset (10) and 6 HCC-related microarrays from the Gene Expression Omnibus (GEO) database (11) were analyzed in combination to obtain differentially expressed lncRNAs in HCC tissues. F-box and leucine-rich repeat protein 19-antisense RNA 1 (FBXL19-AS1) was filtered to be up-regulated in HCC and was predicted to predominantly exist in the cytosol by the LncLocator database (12). Besides, it was found to be enriched in inflammation and cancer-related pathways through the Gene Set Enrichment Analysis (GSEA). Intriguingly, F-box and leucine rich repeat protein 19 (FBXL19), the complementary gene of FBXL19-AS1, was also up-regulated in HCC and was found to be negatively correlated with the amount of macrophage M1 in the high FBXL19 group of HCC tissues. In addition, FBXL19-AS1 functions as a ceRNA has been widely documented in various cancer studies except HCC (13–18). Therefore, we speculated that FBXL19-AS1 might influence the occurrence and development of HCC through the following two pathways: (1) FBXL19-AS1 stabilized FBXL19 to reduce the amount of macrophage M1, and then promoted the occurrence and development of HCC. (2) FBXL19-AS1 might participate in regulating HCC related pathways through FBXL19-AS1-miRNA-mRNA network as ceRNA or inducible endogenous lncRNA. Our study constructed a functional network of FBXL19-AS1 and demonstrated the significance of FBXL19-AS1 in the early diagnosis and prognosis of HCC.

## Materials and Methods

### Data Source

A part of datasets was obtained from GEO database. We performed a comprehensive retrieve to obtain the publicly available HCC non-coding RNA datasets from GEO database (https://www.ncbi.nlm.nih.gov/geo/). Datasets were enrolled in this study based on the following inclusion criteria: (1) datasets contained both normal and HCC samples; (2) HCC subjects were pathologically diagnosed based on clinical and histopathological criteria and without limitation on the clinicopathological stage; (3) type of datasets was non-coding RNA profiling by array; (4) datasets contained more than 5000 lncRNAs. Based on the criteria above, we screened out 6 HCC datasets from GEO database, including GSE58043, GSE67260, GSE112613, GSE89186, GSE64631, and GSE70880. Considering that the limited sample size of a single dataset could lead to unreliable results and reduce the effectiveness of bioinformatics analysis, we integrated all samples of 6 datasets to significantly increase the sample size (39 normal samples vs 44 HCC samples). Heterogeneity and potential variables are generally recognized as the main sources of bias and variability in high-throughput experiments. The merged data were preprocessed by SVA (19) with R software (Version 3.5.3) to remove the batch effect and heterogeneity among various datasets. If a gene corresponded to multiple probes, we took the average as its expression value. Differentially expressed lncRNAs were identified using the limma package (20) in R software and the threshold was |log2(foldchange) | > 1, adjusted *p* value < 0.05.

The other part of data was from TCGA LIHC dataset (https://portal.gdc.cancer.gov/), which contained 50 normal samples and 374 tumor samples. Differentially expressed lncRNAs were filtered using the edgeR package (21) in R software and the threshold was |log2(foldchange)| > 1, adjusted *p* value < 0.05. Details of each dataset were shown in **Table S1**.

### GSEA

GSEA is a bioinformatics method that inspects the statistical significance of *a priori* defined sets of genes and verifies the differences between two biological states (22). Samples from TCGA were divided into 2 subgroups on the basis of the median expression of FBXL19-AS1(NR_024348.1). Genes from each sample were ranked according to the expression difference between the 2 subgroups by GSEA software 4.0. The KEGG gene set was analyzed to explore pathways enriched in each subgroup. Gene set permutations were executed for 1000 times in the analysis. Normalized *p* value < 0.05 was taken as the threshold.

### Tumor Infiltrating Immune Cells Reckoning

CIBERSORT provides a deconvolution algorithm that is able to distinguish 22 kinds of tumor infiltrating immune cells (TIICs) from other cell types in tissues (23). Expression profiles of 50 normal liver tissues and 374 LIHC tumor tissues were downloaded from TCGA database and TIICs proportions of each sample were evaluated by R software on the basis of CIBERSORT algorithm. With *p* value < 0.05 as the threshold, samples were filtered to increase the reliability of the results. Then TIICs proportions of LIHC tumor tissues were divided into 2 subgroups respectively based on the median of FBXL19 expression level and visualized through violin plots.

### Tissue and Plasma Samples

Surgical specimens were obtained from 57 HCC patients (52 males and 5 females) in Zhongnan Hospital of Wuhan University (Wuhan, China) from 2015 to 2019. None of the patients received preoperative chemotherapy or radiotherapy. The follow-up period ranged from 2 months to 48 months. Whole blood samples were collected during 2017 and 2019 from Zhongnan Hospital of Wuhan University, which contained 79 healthy people, 77 patients with hepatitis B, 80 patients with cirrhosis, and 92 patients with HCC. All whole blood samples were collected into the EDTA anticoagulant tubes and the plasma was isolated at 12,000 g for 5min at 4℃. Tissue and plasma samples were stored at -80℃ until use. All patients were diagnosed based on their pathological reports. The tumor stages were identified according to the seventh edition of the American Joint Committee on Cancer (AJCC) Cancer Staging Manual. The detailed clinicopathological information of all participants was shown in **Tables 1** and **2**. All experimental schemes were approved by the Ethics Committee of Zhongnan Hospital of Wuhan University.

**Table 1:** Relationship between FBXL19-AS1 expression in tilssues and clinical characteristics of hepatocellular carcinoma (HCC) patients.

Characteristics	Patient number (n = 57)	Fold change (%)		*p* value
		Low (n = 28)	High (n = 29)	
Gender:				1.000
Female	4	2	2	
Male	53	26	27	
Age (y):				0.889
< 55	30	15	15	
≥ 55	27	13	14	
Smoking:				0.705
Positive	34	16	18	
Negative	23	12	11	
Alcoholism:				0.503
Positive	27	12	15	
Negative	30	16	14	
Tumor size (cm)				0.227
<5	20	12	8	
≥5	37	16	21	
Tumor nodes:				1.000
Single	52	26	26	
Multi	5	2	3	
TNM:				**0.043**
I/II	21	14	7	
III/IV	36	14	22	
Histologic grade:				0.275
Well and moderate	49	26	23	
Low	8	2	6	
ALT (U/l):				0.223
<46	32	18	14	
≥46	25	10	15	
AST (U/l):				0.083
<46	30	18	12	
≥46	27	10	17	
GGT (U/l):				**0.046**
<55	23	15	8	
≥55	34	13	21	
AFP (ng/l):				**0.020**
<200	34	21	13	
≥200	23	7	16	
CEA:				0.967
<5	52	25	27	
≥5	5	3	2	
HBV DNA (IU/ml):				0.190
<500	19	7	12	
≥500	38	21	17	

ALT, alanine aminotransferase; AST, aspartate aminotransferase; GGT, γ-Glutamyl Transferase; AFP, alpha-fetoprotein; CEA, carcinoembryonic antigen; HBV, hepatitis B virus.

Entries in bold font indicate statistically significant (p < 0.05).

**Table 2:** The main clinical features of research subjects.

Characteristics	HCC	Cirrhosis	Hepatitis B	Control	*p* value
	N=92	N=80	N=77	N=79	
Gender:					0.317
Male	72	54	58	54	
Female	20	26	19	25	
Age (y):					0.178
<55	37	25	35	37	
≥55	55	55	42	42	
ALT (U/I)	49.00 (28.75–76.25)	26.00 (20.00–36.00)	88.00 (42.00–252.00)	18.00 (14.00–33.00)	**<0.001**
AST (U/I)	57.50 (33.00–146.25)	48.50 (36.25–61.00)	51.00 (38.00–124.00)	22.00 (18.00–28.00)	**<0.001**
TP (g/l)	63.50 (59.55–70.40)	61.65 (55.55–68.20)	63.40 (56.00–68.70)	68.30 (59.25–73.10)	0.052
ALB (g/l)	35.40 (31.50–39.10)	32.60 (25.50–38.35)	35.00 (31.10–41.50)	44.80 (43.05–46.65)	**<0.001**
GGT (U/I)	31.00 (21.75–64.75)	35.50 (19.75–92.50)	35.00 (21.00–65.00)	28.00 (20.00–46.50)	0.227
ALP (U/I)	119.00 (85.50–216.00)	151.00 (101.00–213.00)	137.00 (93.50–152.50)	88.00 (75.75–153.00)	0.351
GLU (mmol/I)	4.94 (4.56–6.25)	5.16 (4.87–5.76)	4.89 (4.48–5.25)	5.10 (4.61–5.73)	0.700
AFP (ng/ml)	17.46 (2.92–310.48)	4.76 (2.25–14.57)	6.00 (2.41–26.11)	3.10 (1.99–4.53)	**<0.001**
CEA (ng/ml)	2.05 (1.36–2.61)	2.09 (1.35–2.74)	1.96 (1.39–2.63)	1.79 (1.22–2.44)	0.394

TP, total protein; ALB, albumin; ALP, alkaline phosphatase; GLU, glucose.

Entries in bold font indicate statistically significant (p < 0.05).

### RNA Extraction and Quantitative Real-Time PCR

Total RNA was isolated from tissues by TRIZOL reagent (Invitrogen, USA), and RNA from plasma was extracted by RNA Separate Extraction Kit (Bioteke, China). NanoDrop 2000C (Thermo Fisher Scientific, USA) was applied to evaluate the concentration and purity of extracted RNA. Then ReverTra Ace qPCR RT Master Mix with gDNA Remover (Toyobo, Japan) was used to reversely transcribed RNA into complementary DNA (cDNA) at 37℃ for 15min, 50℃ for 5min, and 98℃ for 5min. The quantitative real-time PCR (qPCR) was carried out using SYBR Green I UltraSYBR Mixture (CWBIO, China) on Bio-Rad CFX96 (Bio-Rad Laboratories, USA). We used glyceraldehyde 3-phosphate dehydrogenase (GAPDH) as an endogenous reference gene to normalize the expression level among multiple samples. The specific sequences of each pair of primers were available in **Table S2**. Relative gene expression status was calculated by 2^-ΔCq^. All experiments were repeated twice to intensify the credibility.

### Survival Analysis

Gene Expression Profiling Interactive Analysis2 (GEPIA2, http://gepia2.cancer-pku.cn/) is an online tool for gene expression and survival analysis based on tumor and normal samples from TCGA and GTEx Databases (24). Overall survival was evaluated with the Kaplan-Meier method and compared by the log-rank test on GEPIA2. We set the dataset as LIHC and retrieved the overall survival of FBXL19-AS1 to obtain the information on the relationship between FBXL19-AS1 expression and prognosis of HCC. To further verify the results, 57 HCC patients from Zhongnan Hospital of Wuhan University were followed up and survival analysis was performed on the basis of FBXL19-AS1 expression status.

### Prediction of miRNAs

MiRcode V11 (http://www.mircode.org/) was used to predict miRNAs that would interact with FBXL19-AS1. The highly conserved microRNA families file was downloaded from the miRcode V11 website, and R software was used to predict the complementary miRNAs of FBXL19-AS1. Simultaneously, edgeR package in R software was used to screen out the differentially expressed miRNAs (*p* < 0.05) in HCC based on TCGA. Then we took the intersection of the 2 miRNA lists so as to screen out miRNAs that were both interacted with FBXL19-AS1 and differentially expressed in HCC.

### MiRNA Expression Verification

To further enhance the credibility of the differential expression of the predicted target miRNAs in HCC, we retrieved studies that contained miRNA expression data from TCGA and GEO for verification. All studies included both HCC and normal samples and the sample size of each subgroup was no less than 3. Data were extracted from each study as follows: first author, year of publication, region, data source, platform, number of cases, miRNA ID, and miRNA expression level. The combined standard mean difference (SMD) and 95% confidence interval (95% CI) were calculated by STATA 15.0 (STATA Corp, USA). Compared with the normal control, SMD > 0 indicates that miRNA is up-regulated in HCC samples, while SMD < 0 indicates that miRNA is down-regulated in HCC samples. The statistically significant threshold of two-sided *p* value was set at 0.05.

### MiRNA Targets Prediction

To ensure the miRNA-mRNA interactions conserved in essential cancer pathways, target genes of miRNAs supported simultaneously by miRDB, miRTarBase, and TargetScan were selected by R software. Meanwhile, edgeR package in R software was used to screen out the differentially expressed mRNAs (*p* < 0.05) in HCC based on TCGA. In addition, genes co-expressed with FBXL19-AS1 were obtained from cBioPortal database (*p* < 0.05). Finally, the intersection of the 3 lists was taken as the final target mRNAs.

### Establishment of lncRNA-miRNA-mRNA Expression Network

We constructed a FBXL19-AS1-miRNA-mRNA network and Cytoscape 3.7.2 software was used to visualize the network.

### PPI Network Construction and Hub Genes Selection

Analysis of PPI network is helpful in systematically studying the molecular mechanism of diseases and finding new drug targets. In our study, STRING (V11.0) (https://string-db.org/) (25) was adopted to establish a PPI network, and 0.4 was used as the threshold for interaction score. Subsequently, 12 kinds of algorithms (MCC, DMNC, MNC, Degree, EPC, BottleNeck, EcCentricity, Closeness, Radiality, Betweenness, Stress, ClusteringCoefficient) were jointly used to identify hub genes through Cytoscape 3.7.2 software (26). We took genes whose sum algorithm scores were more than 10,000 to construct hub gene network.

### Functional Enrichment Analysis of Hub Genes

R software and clusterProfiler package (27) were used to execute the Gene Ontology (GO) and Kyoto Encyclopedia of Genes and Genomes (KEGG) enrichment analysis (28, 29). Subsequently, the ggplot2 package was used to visualize the results, and the cut-off value of statistical significance was set to *p* < 0.05.

### Statistical Analysis

All statistical analyses of this research were conducted through Stata SE15 (Stata Corporation, USA), SPSS version 25.0 (SPSS Inc., USA) and GraphPad Prism 8.0 (GraphPad Inc., USA). Mean ± standard deviation (SD) was used to describe the continuous variables of normal distribution. Median and quartile was used to describe the continuous variable of skewed distribution. The paired sample t test or Kruskal-Wallis test were utilized to compare the differences between the two groups. The chi-square test or Fisher’s exact test was used to compare the categorical variables among groups. Correlation analysis was performed by Pearson or Spearman’s test. Receiver operation curve (ROC) was used to evaluate the diagnostic value. The best cutoff point for sensitivity and specificity was selected by the Jorden index. Overall survival was evaluated by Kaplan-Meier method and compared by log-rank test. The cutoff value of statistical significance was set as *p* < 0.05.

## Results

### The Long Noncoding RNA Expression Profile of HCC

**Figure 1:**
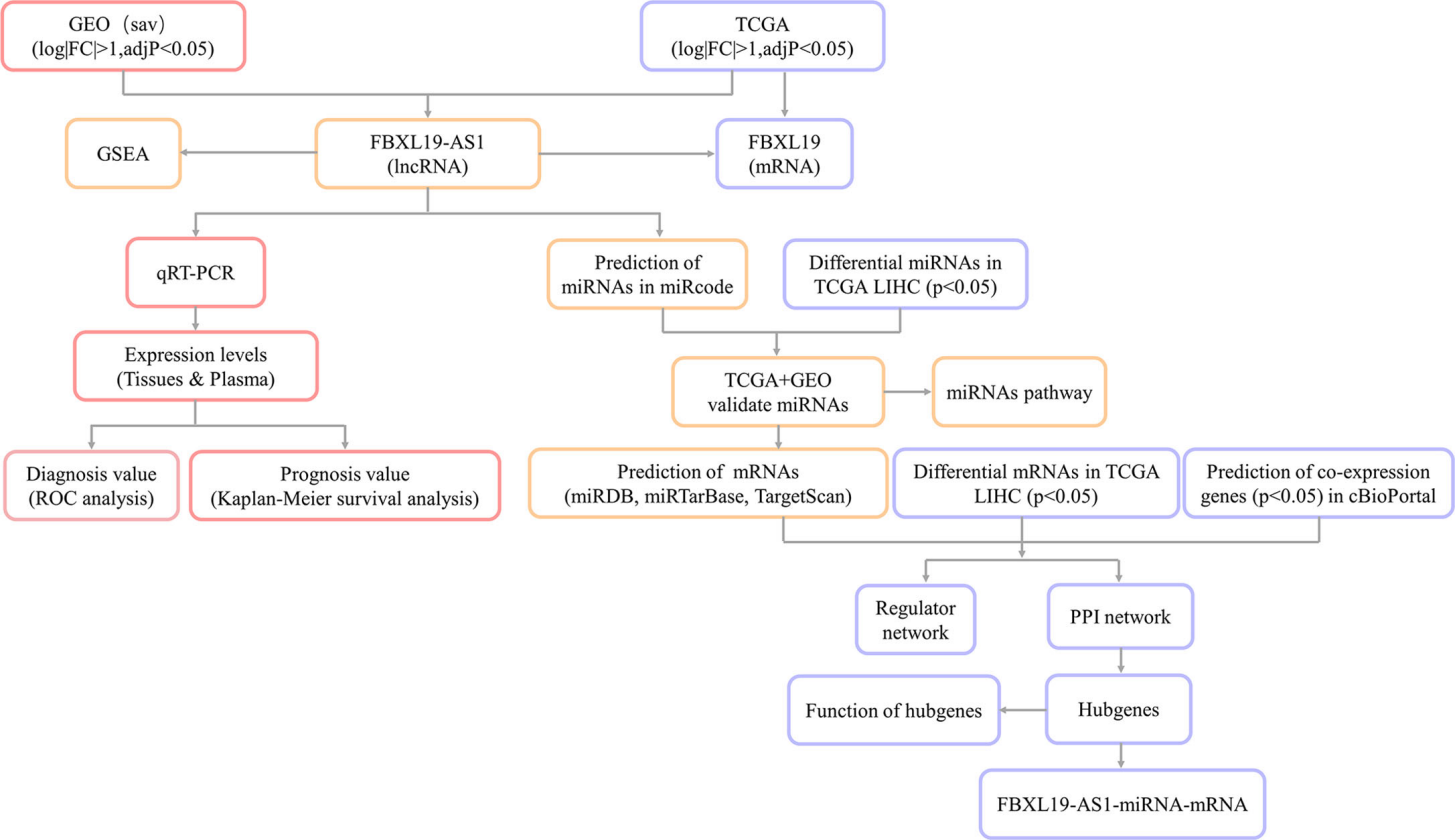
Flow diagram of the analysis process.

**Figure 2:**
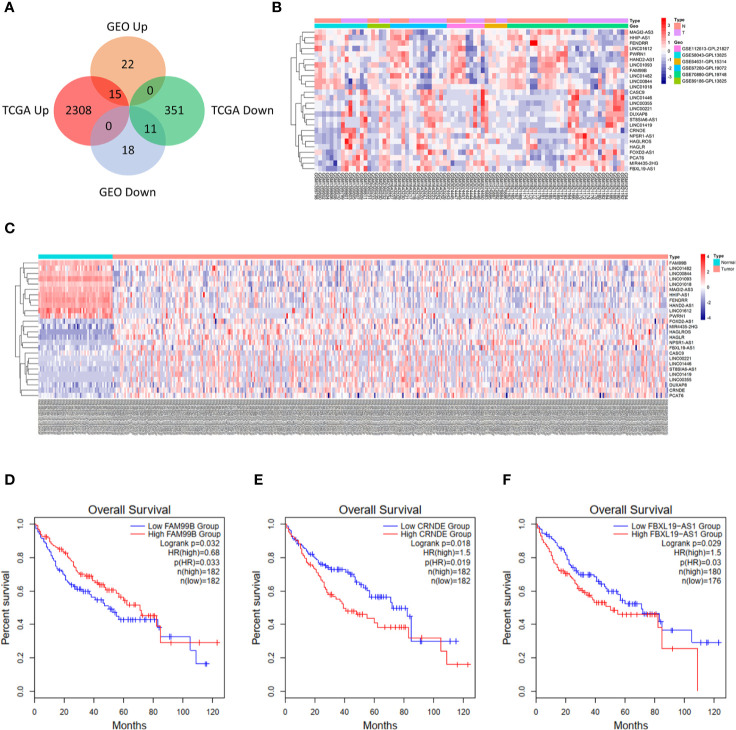
Identification of differentially expressed lncRNAs in hepatocellular carcinoma (HCC). **(A)** Venn diagram for differentially expressed lncRNAs in Gene Expression Omnibus (GEO) joint dataset and The Cancer Genome Atlas liver hepatocellular carcinoma (TCGA LIHC) dataset. **(B)** Heatmap of 26 lncRNAs in GEO joint dataset. **(C)** Heatmap of 26 lncRNAs in TCGA LIHC dataset. **(D)** Overall survival of FAM99B in HCC. **(E)** Overall survival of CRNDE in HCC. **(F)** Overall survival of FBXL19-AS1 in HCC.

The research flow diagram of this study was shown in **Figure 1**. To identify lncRNAs that were differentially expressed in HCC, 6 GEO datasets were integrated into analysis (39 normal samples vs 44 HCC samples), including GSE58043, GSE67260, GSE112613, GSE89186, GSE64631, and GSE70880. We obtained 66 differentially expressed lncRNAs, among which 37 were up-regulated and 29 were down-regulated. Then we downloaded the relevant expression profiles of the TCGA LIHC dataset (50 normal samples vs 374 HCC samples) and obtained 2,685 differentially expressed lncRNAs, among which 2,323 were up-regulated and 362 were down-regulated. There were 26 lncRNAs that were differentially expressed in both the GEO joint dataset and TCGA LIHC dataset, among which 15 were up-regulated and 11 were down-regulated (**Figure 2A**, **Table S3**). The expression heatmaps of the GEO joint dataset and TCGA LIHC dataset were displayed in **Figures 2B, C**, respectively.

The potential interactive miRNAs of 26 differentially expressed lncRNAs were screened by R software based on the highly conserved microRNA families file downloaded from the miRcode V11 database. R software predicted the target genes of corresponding miRNAs that were simultaneously supported by miRDB, miRTarBase and TargetScan. As shown in **Figure S1**, only 7 lncRNAs (LINC00221, FAM99B, LINC00355, MAGI2-AS3, CRNDE, PWRN1, FBXL19-AS1) were predicted to have highly conserved targeted miRNAs. We used GEPIA2 to carry out survival analyses for these 7 lncRNAs, and found that only FBXL19-AS1 (**Figure 2D**), FAM99B (**Figure 2E**), and CRNDE (**Figure 2F**) were associated with the prognosis of HCC patients. Among the 3 lncRNAs, the function of FBXL19-AS1 in HCC has not yet been studied and FBXL19-AS1 might serve as a novel HCC biomarker. Therefore, FBXL19-AS1 was selected for further study.

**Figure 3:**
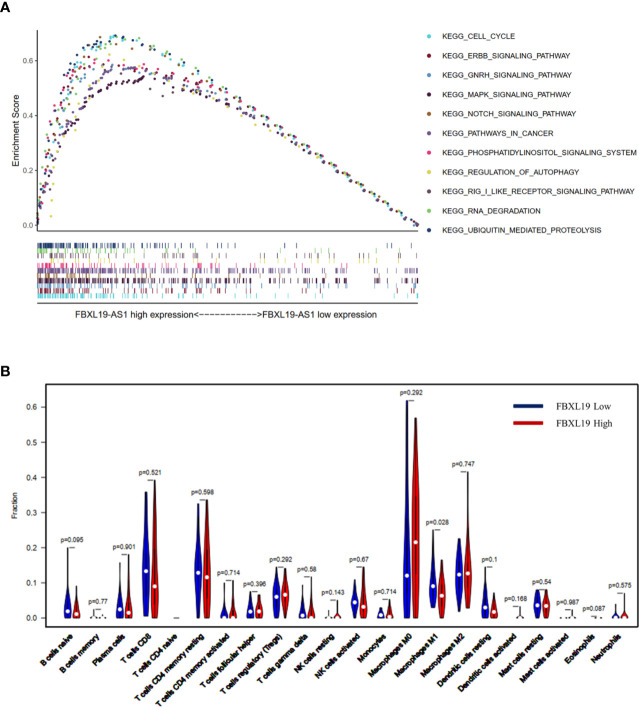
Gene Set Enrichment Analysis (GSEA) analysis on FBXL19-AS1 and relationship between FBXL19 and tumor infiltrating immune cells (TIICs). **(A)** GSEA analysis on FBXL19-AS1. **(B)** Relationship between FBXL19 and TIICs.

FBXL19-AS1 was significantly up-regulated in both the GEO joint dataset and TCGA LIHC datasets and was mainly enriched on cell cycle, cancer and inflammation-related pathways by GSEA (**Figure 3A**). We speculated that FBXL19-AS1 might play an important role in the occurrence, development and prognosis of HCC. In addition, we found that FBXL19-AS1 was located in cytosol according to the LncLocator database (**Figure S2**).

### Relationship Between FBXL19 and TIICs

FBXL19, the complementary gene of FBXL19-AS1, was also up-regulated in HCC and found to be negatively correlated with macrophage M1 (*p* = 0.028) in the high FBXL19 group of HCC tissues base on TCGA LIHC dataset (**Figure 3B**). This result suggested that FBXL19 might reduce the amount of macrophage M1 to influence the tumor immune microenvironment, and then promote the occurrence and development of HCC.

### Expression of FBXL19-AS1 in HCC Tissues and Its Prognostic Value

**Figure 4:**
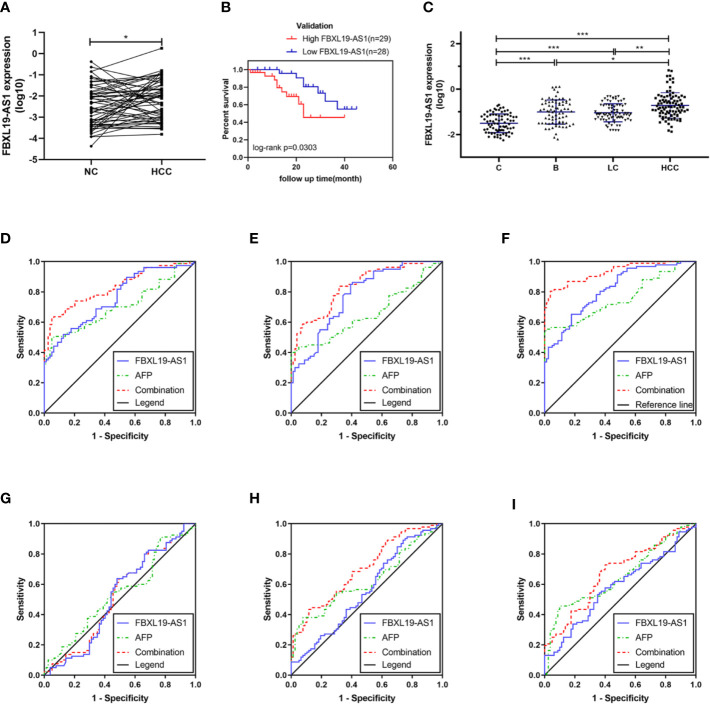
Expression of FBXL19-AS1 and its prognostic and diagnostic value. **(A)** The expression levels of FBXL19-AS1 in hepatocellular carcinoma (HCC) tissues. **(B)** Overall survival of HCC patients with high (n = 29) or low (n = 28) FBXL19-AS1 levels in HCC tissues. **(C)** FBXL19-AS1 expression levels in plasma among the healthy control, hepatitis B, cirrhosis, and HCC groups. Data are presented as mean ± SD. **(D–I)** Receiver operation curves (ROCs) of FBXL19-AS1 and alpha-fetoprotein (AFP) in HCC. **(D)** Healthy control versus hepatitis B. **(E)** Healthy control versus cirrhosis. **(F)** Healthy control versus HCC. **(G)** Hepatitis B versus cirrhosis. **(H)** Hepatitis B versus HCC. **(I)** Cirrhosis versus HCC. *p < 0.05, **p < 0.01 and ***p < 0.001.

In order to verify the expression status of FBXL19-AS1 in HCC patients and to study its clinical significance, we collected 57 pairs of fresh tissues including HCC and adjacent non-tumor tissues. The qPCR results showed that the expression of FBXL19-AS1 in HCC tissue was significantly higher than that in adjacent non-tumor tissue (**Figure 4A**).

According to the median expression level of FBXL19-AS1, HCC samples were divided into low expression group (n = 28) and high expression group (n = 29). The analyses results of the correlation between FBXL19-AS1 expression and clinicopathological features showed that the over expression of FBXL19-AS1 was significantly correlated with higher GGT (*p* = 0.046), higher AFP (*p* = 0.020), and advanced TNM stage (*p* = 0.043), which was consistent with what we predicted in GEPIA2 (**Table 1**, **Figure S3**).

To further validate the prognostic value of FBXL19-AS1 in HCC, we performed Kaplan-Meier survival analysis and log-rank test in 57 HCC patients with intact prognostic information. The results showed that HCC patients with elevated FBXL19-AS1 had shorter overall survival (*p* = 0.030) (**Figure 4B**), hinting FBXL19-AS1 might be an important prognostic factor for HCC.

### Expression of FBXL19-AS1 in Plasma and Its Diagnostic Value

The FBXL19-AS1 expression level was also evaluated through qPCR in plasma samples, which were taken from 79 healthy subjects, 77 patients with hepatitis B, 80 patients with cirrhosis, and 92 patients with HCC (**Figure 4C**). The results showed that the expression of FBXL19-AS1 in plasma was significantly higher in hepatitis B, cirrhosis, and HCC patients than in healthy subjects (p < 0.001). While the expression of FBXL19-AS1 in HCC patients was higher than that in hepatitis B patients (p = 0.016) and cirrhosis patients (p = 0.004). The main clinical features of the plasma samples were shown in **Table 2**. The expression of FBXL19-AS1 was correlated with plasma alanine aminotransferase (ALT) (p < 0.001), aspartate aminotransferase (AST) (p < 0.001), albumin (ALB) (p < 0.001), and alpha fetoprotein (AFP) (p < 0.001), but not with gender, age, CEA, or other biochemical indicators.

ROCs of FBXL19-AS1 in HCC, drawn to evaluate the diagnostic value, indicated that FBXL19-AS1 had moderate diagnostic ability to distinguish HCC patients from healthy people (AUC = 0.875, *p* < 0.001). The predictive value of AFP, the most commonly detected biomarker for the diagnosis of HCC, was rather low (AUC = 0.769, *p* < 0.001). The predictive validity was significantly improved when integration with FBXL19-AS1 (AUC = 0.931, *p* < 0.001). (**Table 3**, **Figures 4D–I**).

**Table 3:** ROC analysis of FBXL19-AS1 and alpha-fetoprotein (AFP) for subgroups.

Subgroup	Biomarker	AUC	95% CI	*p* value	Se (%)	Sp (%)
Controls vs hepatitis B	FBXL19-AS1	0.761	0.688–0.835	**<0.001**	51.95	87.34
	AFP	0.716	0.634–0.798	**<0.001**	49.35	94.94
	Combination	0.831	0.767–0.895	**<0.001**	62.34	94.94
Controls vs cirrhosis	FBXL19–AS1	0.776	0.705–0.846	**<0.001**	85.00	60.76
	AFP	0.668	0.582–0.753	**<0.001**	40.00	100.00
	Combination	0.836	0.775–0.896	**<0.001**	83.75	68.35
Controls vs HCC	FBXL19–AS1	0.875	0.825–0.924	**<0.001**	76.09	82.28
	AFP	0.769	0.699–0.839	**<0.001**	54.35	100.00
	Combination	0.931	0.895–0.967	**<0.001**	80.43	96.20
Hepatitis B vs cirrhosis	FBXL19–AS1	0.482	0.389–0.574	0.694	86.25	29.87
	AFP	0.460	0.370–0.551	0.390	37.50	70.13
	Combination	0.524	0.432–0.616	0.598	63.75	50.65
Hepatitis B vs HCC	FBXL19–AS1	0.635	0.551–0.719	**0.003**	94.57	29.87
	AFP	0.624	0.540–0.708	**0.006**	38.04	92.21
	Combination	0.702	0.624–0.779	**<0.001**	43.48	88.31
Cirrhosis vs HCC	FBXL19–AS1	0.672	0.592–0.752	**0.001**	72.83	60.00
	AFP	0.654	0.572–0.735	**<0.001**	45.65	90.00
	Combination	0.673	0.593–0.753	**<0.001**	72.83	60.00

AUC, area under the curve; Se, sensitivity; Sp, specificity.

Entries in bold font indicate statistically significant (p < 0.05).

### lncRNA-miRNA Interaction Prediction

**Figure 5:**
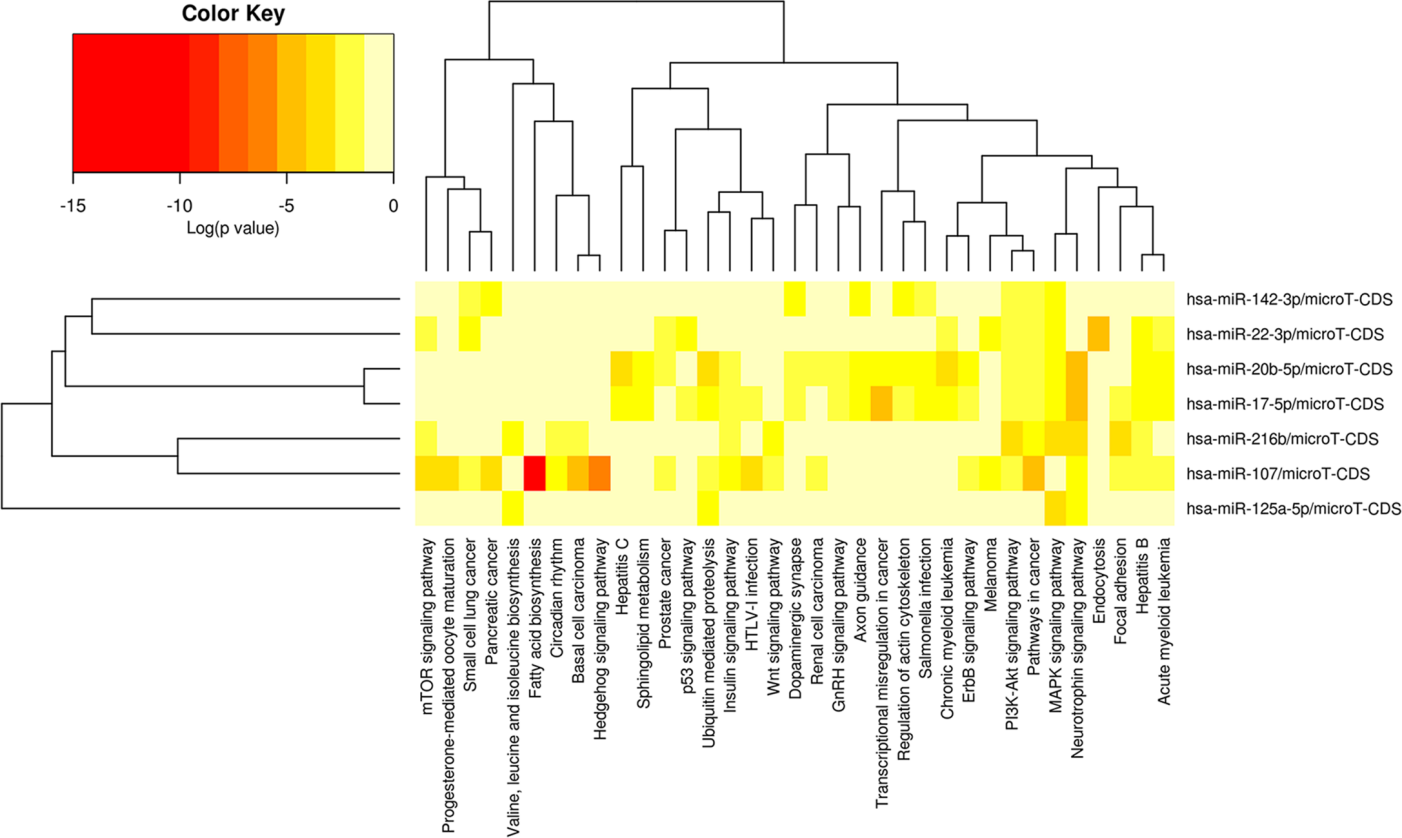
Heatmap for the signaling pathways from DIANA-miRPath in which the 7 miRNAs are involved.

78 miRNAs were obtained as the potential target miRNAs of FBXL19-AS1 through binding site prediction according to miRcode V11 database. 24 miRNAs were selected after comparing with the 467 differentially expressed miRNAs screened from TCGA (p < 0.05). Subsequently, miRNA target genes were predicted and only 7 of the above 24 miRNAs met our requirements (hsa-miR-142-3p, hsa-miR-125a-5p, hsa-miR-216b-5p, hsa-miR-107, hsa-miR-17-5p, hsa-miR-20b-5p, hsa-miR-22-3p). Afterwards, we explored signaling pathways related with the 7 miRNAs through DIANA-miRPath. As shown in **Figure 5**, the 7 miRNAs were involved in the initiation and progression of many types of cancers. Among them, pathways that might be related to HCC including mTOR signaling pathway, Hedgehog signaling pathway, hepatitis B, hepatitis C, p53 signaling pathway, Wnt signaling pathway, GnRH signaling pathway, ErbB signaling pathway, PI3K-Akt signaling pathway, MAPK signaling pathway, and so on.

### Validation of miRNA Expression Based on GEO and TCGA Databases

**Figure 6:**
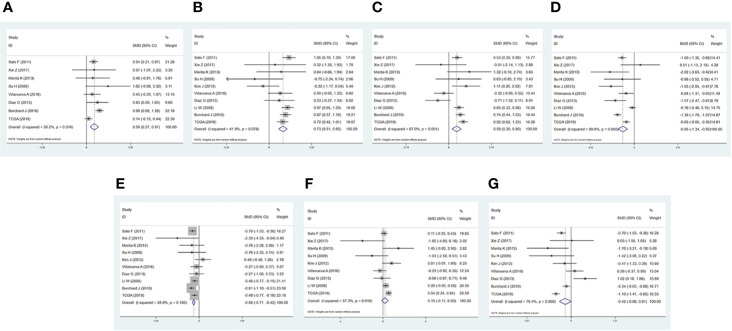
Forest diagrams of datasets assessing the levels of the predicted miRNAs in hepatocellular carcinoma (HCC). **(A)** has-miR-216b-5p. **(B)** has-miR-107. **(C)** has-miR-17-5p. **(D)** has-miR-125a-5p. **(E)** has-miR-22-3p. **(F)** has-miR-20b-5p. **(G)** has-miR-142-3p.

To evaluate the expression of these 7 miRNAs in HCC, 9 GSE microarrays from GEO and dataset from TCGA were selected for verification (**Table S4**). Compared with normal liver tissues, hsa-miR-216b-5p (SMD = 0.593, p = 0.000, **Figure 6A**), hsa-miR-107 (SMD = 0.729, p = 0.000, **Figure 6B**), hsa-miR-17-5p (SMD = 0.502, p = 0.001, **Figure 6C**) were up-regulated in HCC tissues, while hsa-miR-125a-5p (SMD = -0.947, p = 0.000, **Figure 6D**), hsa-miR-22-3p (SMD = -0.563, p = 0.000, **Figure 6E**) were down-regulated in HCC tissues. In addition, hsa-miR-20b-5p (SMD = 0.194, p = 0.217, **Figure 6F**), and hsa-miR-142-3p (SMD = -0.425, p = 0.056, **Figure 6G**) were not differentially expressed between normal tissues and HCC tissues, more studies with larger sample sizes were still needed.

**Figure 7:**
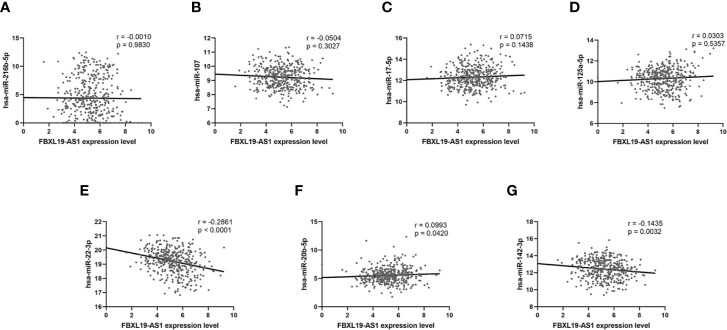
The correlation analysis between FBXL19-AS1 and 7 miRNAs. **(A)** has-miR-216b-5p. **(B)** has-miR-107. **(C)** has-miR-17-5p. **(D)** has-miR-125a-5p. **(E)** has-miR-22-3p. **(F)** has-miR-20b-5p. **(G)** has-miR-142-3p.

We analyzed the correlation between the expression of FBXL19-AS1 and these 7 miRNAs based on the TCGA LIHC dataset. Significant correlations were not found in hsa-miR-216b-5p (r = -0.0010, *p* = 0.9830, **Figure 7A**), hsa-miR-107 (r = -0.0504, *p* = 0.3027, **Figure 7B**), hsa-miR-17-5p (r = 0.0715, *p* = 0.1438, **Figure 7C**), or hsa-miR-125a-5p (r = 0.0303, *p* = 0.5357, **Figure 7D**), but the relations were prominent in hsa-miR-22-3p (r = -0.2861, *p* < 0.001, **Figure 7E**), hsa-miR-20b-5p (r = 0.0993, *p* = 0.0420, **Figure 7F**), and hsa-miR-142-3p (r = -0.1435, *p* = 0.0032, **Figure 7G**). Hence, we took hsa-miR-22-3p, hsa-miR-20b-5p and hsa-miR-142-3p for subsequent analyses.

### LncRNA-miRNA-mRNA Network Construction

To further explore the potential downstream targets of these 3 miRNAs, three online bioinformatics servers (miRDB, miRTarBase, and TargetScan) were used. There were 399 target genes of these 3 miRNAs simultaneously supported by all three databases. Then, we screened out 12,841 differentially expressed mRNAs from TCGA LIHC dataset (p < 0.05). In addition, 12,194 mRNAs were predicted to be co-expressed with FBXL19-AS1 in HCC by cBioportal database (p < 0.05). Finally, 205 mRNAs were selected as targets through the intersection of the above three gene sets.

**Figure 8:**
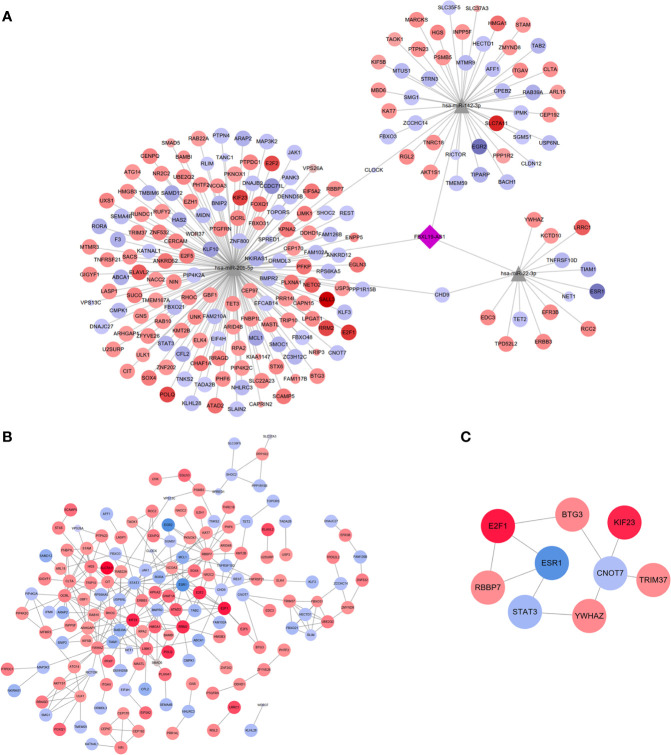
LncRNA-miRNA-mRNA network, PPI network and hub genes network. **(A)** The network including lncRNAs (FBXL19-AS1), 3 miRNAs (hsa-miR-22-3p, hsa-miR-20b-5p, hsa-miR-142-3p) and 205 mRNAs. **(B)** PPI network on these 205 mRNAs. **(C)** Hub genes network contained 9 nodes and 9 edges.

A new FBXL19-AS1-miRNA-mRNA network was formed among FBXL19-AS1, three miRNAs (hsa-miR-22-3p, hsa-miR-20b-5p, hsa-miR-142-3p) and 205 mRNAs (**Figure 8A**). The diamond in the middle represented FBXL19-AS1, the gray triangles were miRNAs, and the circles were mRNAs. The circles in red meant the corresponding mRNAs were elevated in HCC, blue circles represented the decreased expression of related mRNAs in HCC, deeper colors indicated increased logFC, and larger sizes indicated smaller *p* values.

### PPI Network Construction and Screening of Hub Genes

We constructed a PPI network of these 205 mRNAs based on the STRING database and then visualized by Cytoscape 3.7.2. After removing the free nodes, the PPI network contained 158 nodes and 272 edges (**Figure 8B**). Thereafter, hub genes identified by 12 algorithms (MCC, DMNC, MNC, Degree, EPC, BottleNeck, EcCentricity, Closeness, Radiality, Betweenness, Stress, ClusteringCoefficient) constituted a subnetwork with 9 nodes and 9 edges (**Figure 8C**), which revealed the 9 hub genes (STAT3, CNOT7 BTG3, E2F1, TRIM37, YWHAZ, RBBP7, KIF23, ESR1) played important roles in the pathogenesis of HCC.

### Functional Analysis of 9 Hub Genes

**Figure 9:**
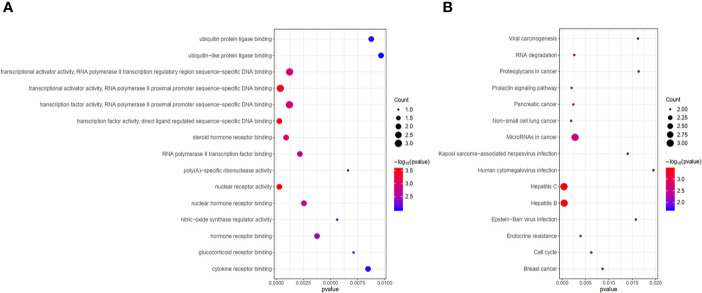
Functional analysis of 9 hub genes. **(A)** Gene ontology (GO) functional enrichment analysis of 9 hub genes. **(B)** KEGG pathway enrichment analysis of 9 hub genes.

GO and KEGG enrichment analyses were performed on the 9 hub genes (p < 0.05). The Top 15 terms and pathways were selected for demonstration by p value. GO functional enrichment analysis revealed hub genes mainly enriched in transcription factor activity and transcriptional activator activity (**Figure 9A**). KEGG pathway analysis indicated that the 9 hub genes might influence the occurrence and progression of HCC by participating in pathways such as hepatitis C, hepatitis B, microRNAs in cancer, cell cycle, viral carcinogenesis, and proteoglycans in cancer (**Figure 9B**). In addition, the 9 hub genes were also involved in pathways associated with non-small cell lung cancer, pancreatic cancer, breast cancer and other diseases.

### Verification of FBXL19-AS1-miRNA-mRNA Network

**Figure 10:**
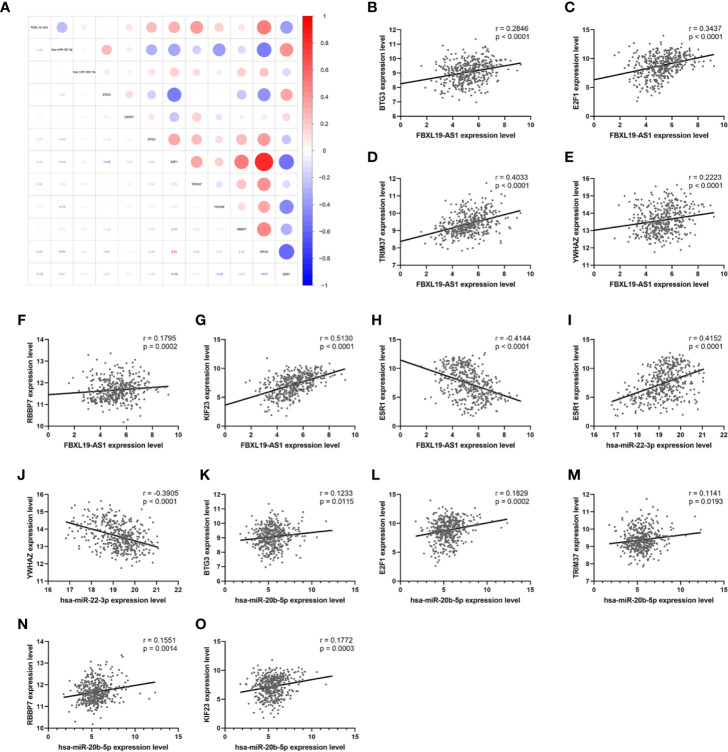
The correlation analysis of FBXL19-AS1, 2 miRNAs, and 9 hub genes. **(A)** Correlation heatmap of the whole network. **(B–O)** The correlation analyses which were statistically significant.

**Figure 11:**
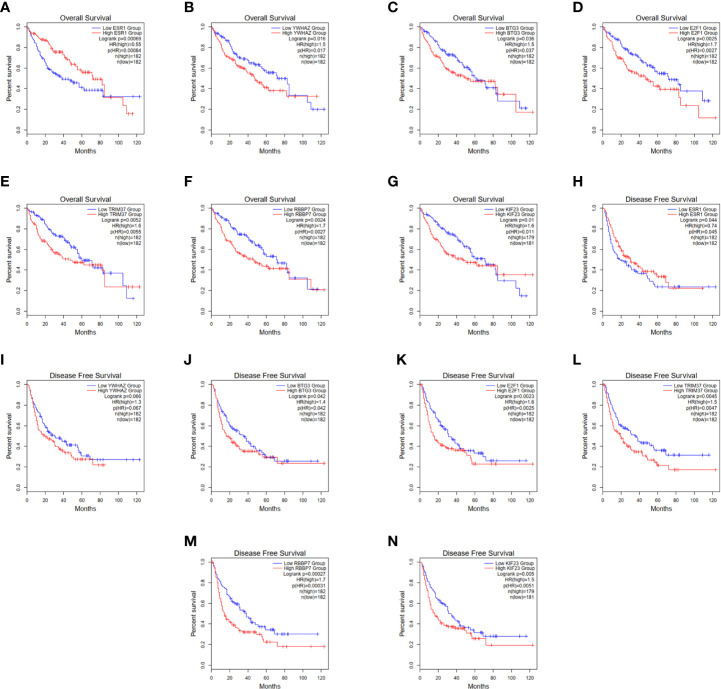
Overall survival and disease-free survival of 7 hub genes. **(A–G)** Overall survival of 7 hub genes. **(H-N)** Disease-free survival of 7 hub genes.

**Figure 12:**
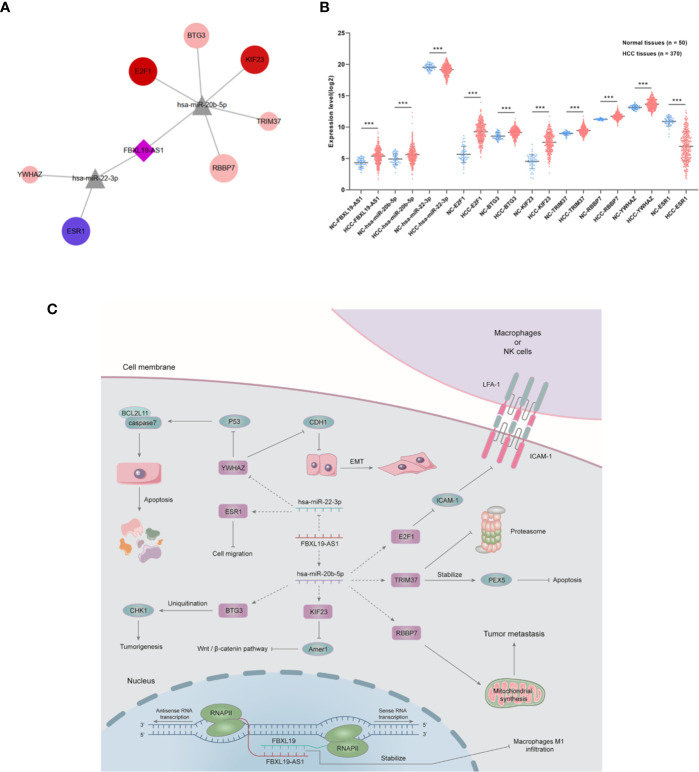
FBXL19-AS1-miRNA-mRNA network. **(A)** The network including 1 lncRNA (FBXL19-AS1), 2 miRNAs (has-miR-22-3p, has-miR-20b-5p), and 7 mRNAs (BTG3, E2F1, TRIM37, YWHAZ, RBBP7, KIF23, ESR1). **(B)** The expression levels of whole FBXL19-AS1-miRNA-mRNA network. **(C)** Functional network of FBXL19-AS1 and its possible downstream pathways in hepatocellular carcinoma. ***p < 0.001.

In order to establish a more reliable FBXL19-AS1-miRNA-mRNA network, we performed correlation analyses among FBXL19-AS1, 2 miRNAs and 9 mRNAs based on 370 HCC tissues and 50 normal tissues from TCGA LIHC dataset. Considering none of the 9 hub genes was correlated with hsa-miR-142-3p, it was not enrolled into the subsequent analyses. Significant correlations were found in FBXL19-AS1, hsa-miR-22-3p, hsa-miR-20b-5p, and 7 mRNAs, except STAT3 and CNOT9 (**Figure 10**). Survival analyses of OS and DFS of the remaining 7 hub genes were performed by the Kaplan-Meier method in the GEPIA2 database. Remarkable survival differences existed in nearly all the 7 hub genes, except the DFS of YWHAZ (**Figure 11**), which indicated all the 7 hub genes were associated with the prognosis of HCC. Therefore, we constructed a FBXL19-AS1-miRNA-mRNA network consisting of FBXL19-AS1, two miRNAs, seven hub genes, and seven lncRNA-miRNA-mRNA regulatory pathways (FBXL19-AS1/miR-22-3p/YWHAZ axis, FBXL19-AS1/miR-22-3p/ESR1 axis, FBXL19-AS1/miR-20b-5p/E2F1 axis, FBXL19-AS1/miR-20b-5p/BTG3 axis, FBXL19-AS1/miR-20b-5p/KIF23 axis, FBXL19-AS1/miR-20b-5p/KIF23 axis, FBXL19-AS1/miR-20b-5p/TRIM37 axis, FBXL19-AS1/miR-20b-5p/RBBP7 axis) (**Figure 12A**). To further verify the reliability of the network, we analyzed the expression status of the elements in the network based on TCGA LIHC dataset. As shown in **Figure 12B**, FBXL19-AS1, hsa-miR-20b-5p, hsa-miR-22-3p, and 7 hub genes were all differentially expressed between normal tissues and HCC tissues.

## Discussion

In our study, FBXL19-AS1 was identified as a potential oncogene through integrated analysis of six GEO microarray datasets and TCGA LIHC dataset. Previous studies have shown that FBXL19-AS1 was significantly increased in breast cancer (13, 14, 30), lung cancer (15, 16, 31), osteosarcoma (17), colorectal cancer (18), cervical cancer (32), and glioma (33), and participated in the migration, proliferation and survival of tumor cells. Dong et al. found that FBXL19-AS1 mainly existed in the cytoplasm and was up-regulated in a variety of breast cancer cell lines. Knockdown FBXL19-AS1 could inhibit cell proliferation and induce cell apoptosis through acting as the sponge of miR-876-5p in breast cancer cells (13). Ding et al. identified the up-regulation of FBXL19-AS1 not only *in vitro*, but also in breast cancer tissues. Besides, FBXL19-AS1 was demonstrated to target miR-718 to promote cell proliferation, invasion and EMT of breast cancer cells (14). Similar phenomena were also observed by Zhang et al. that the overexpression of FBXL19-AS1 in breast cancer cells promoted cell migration and EMT (30). FBXL19-AS1 was also significantly up-regulated in lung adenocarcinoma (LUAD) tissues, and the high expression of FBXL19-AS1 in LUAD was associated with poor prognosis. In addition, FBXL19-AS1 knockdown could arrest LUAD cells from entering G0/G1 phase and inhibit cell proliferation, migration and invasion by affecting miR-203a-3p (16). FBXL19-AS1 was over-expressed and more enriched in the cytoplasm of glioma endothelial cells (GECs). IGF2BP2 knockdown combined with FBXL19-AS1 knockdown increased the effect of doxorubicin in promoting apoptosis of U87 cells (33). The roles that FBXL19-AS1 played in the above-mentioned tumors have been confirmed, the underlying function of FBXL19-AS1 in HCC has not yet been investigated yet. To verify the bioinformatics results and explore the role of FBXL19-AS1 in HCC, qPCR was used to assess the expression status of FBXL19-AS1. The experimental results indicated that FBXL19-AS1 was elevated in HCC and its expression was correlated with TNM stage, AFP and GGT. Given that elevated GGT is associated with the occurrence of acute and chronic hepatitis and alcoholic liver disease, it can be inferred that FBXL19-AS1 may be involved in the development of related diseases. In combination with the GEPIA2 survival analysis results and our follow-up study on 57 HCC patients, we found that high expression of FBXL19-AS1 was associated with poor prognosis in HCC.

Biomarkers screened from liver tissues are not suitable for the early diagnosis of HCC. The specificity and sensitivity of AFP, the most widely used plasma biomarker in HCC diagnosis, are quite limited. To compensate for the deficiency of early diagnosis of HCC, we evaluated the diagnostic value of FBXL19-AS1 in plasma. The results showed that FBXL19-AS1 in plasma of patients with hepatitis B, cirrhosis and HCC was significantly higher than that in healthy subjects. ROC analysis revealed that plasma FBXL19-AS1 had satisfactory diagnostic value in differentiating healthy controls from patients with hepatitis B, cirrhosis, and especially HCC. Whereas, the discernibility ability of FBXL19-AS1 in hepatitis B patients and cirrhosis patients was unsatisfactory, which could be partially explained by the pathological similarity of the patients. It should be noted that the combination of plasma FBXL19-AS1 and AFP could significantly improve the diagnosis for HCC, suggesting that FBXL19-AS1 could serve as a biomarker for the auxiliary diagnosis of HCC. It is also noteworthy that FBXL19-AS1 has been reported to be associated with a variety of cancers, and therefore, the need to combine FBXL19-AS1 and AFP to enhance the diagnostic specificity of HCC should be emphasized.

The result of TIICs showed that up-regulated FBXL19 was negatively correlated with macrophage M1. Macrophages M1 showed tumoricidal activity and promoted T helper (Th) 1 responses (34). Thus, FBXL19-AS1 might reduce the amount of macrophage M1 to restrain the tumoricidal activity by stabilizing the expression of FBXL19, and then promote the occurrence and development of HCC.

In addition, FBXL19-AS1 might participate in regulating HCC related pathways through FBXL19-AS1-miRNA-mRNA network as ceRNA or inducible endogenous lncRNA. The potential interactions between FBXL19-AS1 and 7 miRNAs were revealed through multi-step bioinformatics analyses. Encouragingly, the expression levels of hsa-miR-125a-5p, hsa-miR-107, hsa-miR-17-5p, and hsa-miR-22-3p in HCC were consistent with published studies (35–38). However, several studies have come up with results that were ambivalent with ours in hsa-miR-216b-5p, hsa-miR-142-3p, and hsa-miR-20b-5p (39–41). Since the heterogeneity of cross-studies cannot be ignored and the credibility of the combined results may decrease due to interfusion of the low-quality studies, more studies are needed to verify these results.

In order to elucidate the FBXL19-AS1-miRNA-mRNA network regulatory mechanism, we established a PPI network and obtained 9 hub genes (STAT3, CNOT7 BTG3, E2F1, TRIM37, YWHAZ, RBBP7, KIF23, ESR1). GO functional annotation and KEGG pathway analysis revealed that the 9 hub genes were enriched in hepatitis B associated pathways and the important roles of these 9 hub genes in HCC have also been confirmed in published studies (42–50). Through correlation analysis based on TCGA LIHC tissue samples, STAT3, and CNOT7 were excluded, and the remaining 7 mRNAs were all associated with the prognosis of HCC. Finally, we constructed a more reliable FBXL19-AS1-miRNA-mRNA network consisting of FBXL19-AS1, hsa-miR-22-3p, hsa-miR-20b-5p, 7 hub genes (BTG3, E2F1, TRIM37, YWHAZ, RBBP7, KIF23, ESR1), and 7 lncRNA-miRNA-mRNA regulatory axes. A study reported that miR-22 and miR-20b were involved in the progression of HBV-related HCC, which further improved the credibility of our FBXL19-AS1-miRNA-mRNA network (51).

On the grounds of previous published studies and our research, we integrated the potential regulation mechanisms of FBXL19-AS1 in cancers and visualized the underlying functional network of FBXL19-AS1 in HCC cells (**Figure 12C**). FBXL19-AS1 could form double stranded structure with FBXL19 to stabilize FBXL19 by protecting the mRNA from degradation. Our study indicated the elevated FBXL19 might restrain the infiltration and tumoricidal activity of macrophages M1 in HCC tumor tissues and thus accelerate the development of HCC. E2F1, one of the hub genes of FBXL19-AS1, also participated in the anti-tumoricidal activity by downregulating ICAM-1 and reducing the adhesion of macrophages or NK cells (52). FBXL19-AS1 might act as the inducible endogenous RNA of hsa-miR-20b-5p to influence the expression of BTG3, KIF23, RBBP7, and TRIM37 and regulate tumorigenesis, Wnt/β-catenin pathway, tumor metastasis as well as apoptosis (53–56). FBXL19-AS1 could attenuate the inhibitory of hsa-miR-22-3p on YWHAZ to suppress apoptosis and promote epithelial-mesenchymal transition (EMT) (47). ESR1 has been reported to affect tumor cell migration, the relationship among FBXL19-AS1, hsa-miR-22-3p, and ESR1 needs further verification (57).

## Conclusion

In summary, FBXL19-AS1 was identified as an oncogenic lncRNA that may serve as a diagnostic and prognostic agent for potential HCC biomarkers. FBXL19-AS1 might stabilize FBXL19 to reduce the amount of macrophage M1, and then promote the occurrence and development of HCC. We also established a FBXL19-AS1-miRNA-mRNA network, and demonstrated that FBXL19-AS1 might participate in the pathological progression of HCC through this network. Our study elucidates the potential oncogenic pathways in which FBXL19-AS1 is involved and recognizes the regulatory role of FBXL19-AS1 on the underlying target genes. Our findings may provide new perspectives on the pathogenesis of HCC, thus broadening the therapeutic options for HCC.

## Data Availability Statement

The original contributions presented in the study are included in the article/**Supplementary Material**. Further inquiries can be directed to the corresponding authors.

## Ethics Statement

The studies involving human participants were reviewed and approved by The Ethics Committee of Zhongnan Hospital of Wuhan University. The patients/participants provided their written informed consent to participate in this study.

## Author Contributions

DH and JT designed the workflow and wrote this paper. DH, XKZ, XYZ, and NM performed the experiments, analyzed the data. YW and PL collected the samples. JT and CL revised the manuscript. All authors contributed to the article and approved the submitted version.

## Funding

This research was funded by the National Basic Research Program of China (2012CB720605) and Zhongnan Hospital of Wuhan University Science, Technology and Innovation Seed Fund (ZNPY2017054).

## Conflict of Interest

The authors declare that the research was conducted in the absence of any commercial or financial relationships that could be construed as a potential conflict of interest.
