# Supplementary material for: Identify and Validate the Transcriptomic, Functional Network, and Predictive Validity of FBXL19-AS1 in Hepatocellular Carcinoma
Source: Front Oncol. 2020 Dec 3;10:609601. doi: 10.3389/fonc.2020.609601 (PMC7744744; doi:10.3389/fonc.2020.609601)
Supplement: Supplementary file 1 [file DataSheet_1.zip › Supplementary material/Table S1.docx]

**Table S1** Details of microarray datasets from GEO and TCGA.

| Data source | Platform | Region | Sample type | Sample size (T/N) | Technology |
| --- | --- | --- | --- | --- | --- |
| GSE58043 | GPL13825 | China | paired tissue | 7/7 | Arraystar Human LncRNA microarray V2.0 (Agilent-033010 Feature Number version) |
| GSE89186 | GPL13825 | China | paired tissue | 3/3 | Arraystar Human LncRNA microarray V2.0 (Agilent-033010 Feature Number version) |
| GSE67260 | GPL19072 | China | unpaired tissue | 10/5 | Agilent-052909 CBC_lncRNAmRNA_V3 (Probe Name version) |
| GSE112613 | GPL21827 | China | paired tissue | 5/5 | Agilent-079487 Arraystar Human LncRNA microarray V4 (Probe Name version) |
| GSE64631 | GPL15314 | USA | paired tissue | 3/3 | Arraystar Human LncRNA microarray V2.0 (Agilent_033010 Probe Name version) |
| GSE70880 | GPL19748 | China | paired tissue | 16/16 | Agilent-038314 CBC Homo sapiens lncRNA + mRNA microarray V2.0 (Probe Name version) |
| TCGA | TCGA | USA | unpaired tissue | 374/50 | Illumina HiSeq |
