# Supplementary material for: Identify and Validate the Transcriptomic, Functional Network, and Predictive Validity of FBXL19-AS1 in Hepatocellular Carcinoma
Source: Front Oncol. 2020 Dec 3;10:609601. doi: 10.3389/fonc.2020.609601 (PMC7744744; doi:10.3389/fonc.2020.609601)
Supplement: Supplementary file 1 [file DataSheet_1.zip › Supplementary material/Table S2.docx]

**Table S2** Primer sequence and Tm for qPCR.

| Gene | Primer sequence（5’→3’） | Tm（℃） | Product length（bp） |
| --- | --- | --- | --- |
| FBXL19-AS1 | F: GTCGAGACAATGGAAGGGGA | 60 | 96 |
|  | R: GACGCCTGGACTACACATCC |  |  |
| GAPDH | F: GGTCTCCTCTGACTTCAACA | 60 | 221 |
|  | R: GTGAGGGTCTCTCTCTTCCT |  |  |
