# Supplementary material for: Identify and Validate the Transcriptomic, Functional Network, and Predictive Validity of FBXL19-AS1 in Hepatocellular Carcinoma
Source: Front Oncol. 2020 Dec 3;10:609601. doi: 10.3389/fonc.2020.609601 (PMC7744744; doi:10.3389/fonc.2020.609601)
Supplement: Supplementary file 1 [file DataSheet_1.zip › Supplementary material/Table S3.docx]

**Table S3** Expression of 26 differentially expressed lncRNAs.

| Gene ID | GEO | | TCGA | |
| --- | --- | --- | --- | --- |
|  | logFC | adj.P.Val | logFC | adj.P.Val |
| LINC01419 | 2.300393 | 0.006302 | 10.97693 | 2.89E-19 |
| DUXAP8 | 2.078285 | 8.40E-07 | 2.966888 | 1.26E-17 |
| CASC9 | 2.060969 | 9.14E-05 | 3.128698 | 2.14E-06 |
| LINC00355 | 1.61055 | 0.006302 | 7.16815 | 1.70E-18 |
| ST8SIA6-AS1 | 1.604583 | 0.003864 | 5.533221 | 3.12E-20 |
| MIR4435-2HG | 1.361977 | 1.68E-08 | 1.658091 | 3.54E-18 |
| NPSR1-AS1 | 1.318596 | 0.000177 | 6.256856 | 8.09E-18 |
| FBXL19-AS1 | 1.286586 | 4.43E-05 | 1.432504 | 5.16E-12 |
| CRNDE | 1.252964 | 0.000649 | 3.178178 | 1.50E-22 |
| HAGLROS | 1.181868 | 8.78E-05 | 4.775213 | 2.47E-29 |
| LINC01446 | 1.131583 | 0.02083 | 4.416132 | 2.29E-09 |
| PCAT6 | 1.076771 | 0.000112 | 1.45458 | 2.80E-12 |
| LINC00221 | 1.075629 | 0.047329 | 7.569363 | 6.24E-13 |
| HAGLR | 1.056741 | 0.006302 | 6.091234 | 1.13E-27 |
| FOXD2-AS1 | 1.049399 | 3.64E-05 | 1.943038 | 1.36E-18 |
| PWRN1 | -1.05345 | 0.000144 | -1.65613 | 1.48E-05 |
| LINC01018 | -1.21789 | 0.007097 | -1.60559 | 5.31E-07 |
| LINC01612 | -1.25958 | 4.15E-05 | -3.15088 | 1.43E-11 |
| HAND2-AS1 | -1.26538 | 3.64E-05 | -2.43512 | 2.26E-15 |
| MAGI2-AS3 | -1.29054 | 6.35E-06 | -1.80464 | 9.01E-15 |
| FENDRR | -1.37732 | 2.12E-05 | -2.81553 | 1.92E-28 |
| LINC01482 | -1.39167 | 2.70E-05 | -1.05585 | 1.28E-05 |
| FAM99B | -1.46339 | 0.000451 | -1.81348 | 3.07E-09 |
| HHIP-AS1 | -1.77155 | 2.43E-10 | -1.43301 | 2.49E-05 |
| LINC00844 | -2.0367 | 0.000197 | -1.56468 | 8.22E-06 |
| LINC01093 | -3.34598 | 2.45E-12 | -3.60475 | 1.11E-37 |
