# Supplementary material for: Identify and Validate the Transcriptomic, Functional Network, and Predictive Validity of FBXL19-AS1 in Hepatocellular Carcinoma
Source: Front Oncol. 2020 Dec 3;10:609601. doi: 10.3389/fonc.2020.609601 (PMC7744744; doi:10.3389/fonc.2020.609601)
Supplement: Supplementary file 1 [file DataSheet_1.zip › Supplementary material/Table S4.docx]

**Table S4** Essential information of the studies for the 7 miRNAs derived from GEO and TCGA database.

| miRNA | Study | Year | Region | Data source | Platform | Number of cases | | Expression(mean±SD) | | |
| --- | --- | --- | --- | --- | --- | --- | --- | --- | --- | --- |
|  |  |  |  |  |  | Cancer | Normal | | Cancer | Normal |
| hsa-miR-20b-5p | Sato F | 2011 | Japan | GSE21362 | GPL10312 | 73 | 73 | 8.55±1.282 | | 8.43±0.900 |
|  | Xie Z | 2017 | China | GSE98269 | GPL20712 | 3 | 3 | 8.18±0.646 | | 9.11±0.229 |
|  | Morita K | 2013 | Japan | GSE41874 | GPL7722 | 6 | 4 | 1.29±0.498 | | 0.70±0.166 |
|  | Su H | 2009 | China | GSE12717 | GPL7274 | 5 | 3 | 11.47±0.679 | | 12.08±0.354 |
|  | Kim J | 2012 | South Korea | GSE39678 | GPL15852 | 16 | 8 | 10.92±0.824 | | 10.36±0.228 |
|  | Villanueva A | 2016 | Spain | GSE74618 | GPL14613 | 218 | 10 | 3.04±0.317 | | 3.13±0.224 |
|  | Diaz G | 2013 | USA | GSE40744 | GPL14613 | 9 | 19 | 3.62±0.232 | | 3.64±0.256 |
|  | Li W | 2008 | China | GSE10694 | GPL6542 | 78 | 88 | 12.01±0.953 | | 11.77±0.786 |
|  | TCGA | 2019 | USA | TCGA | TCGA | 375 | 50 | 5.61±1.345 | | 4.91±0.872 |
| hsa-miR-216b-5p | Sato F | 2011 | Japan | GSE21362 | GPL10312 | 73 | 73 | 1.86±2.140 | | 0.96±1.017 |
|  | Xie Z | 2017 | China | GSE98269 | GPL20712 | 3 | 3 | 5.02±0.057 | | 4.99±0.047 |
|  | Morita K | 2013 | Japan | GSE41874 | GPL7722 | 6 | 4 | 1.15±0.192 | | 1.04±0.285 |
|  | Su H | 2009 | China | GSE12717 | GPL7274 | 5 | 3 | 7.72±1.937 | | 5.04±0.825 |
|  | Villanueva A | 2016 | Spain | GSE74618 | GPL14613 | 218 | 10 | 2.16±0.828 | | 1.81±0.297 |
|  | Diaz G | 2013 | USA | GSE40744 | GPL14613 | 9 | 19 | 2.73±0.794 | | 2.34±0.199 |
|  | Burchard J | 2010 | USA | GSE22058 | GPL10457 | 96 | 96 | -0.84±0.411 | | -1.14±0.140 |
|  | TCGA | 2019 | USA | TCGA | TCGA | 375 | 50 | 4.22±4.38 | | 3.62±1.268 |
| hsa-miR-142-3p | Sato F | 2011 | Japan | GSE21362 | GPL10312 | 73 | 73 | 4.39±1.906 | | 5.59±1.507 |
|  | Xie Z | 2017 | China | GSE98269 | GPL20712 | 3 | 3 | 9.48±1.448 | | 9.43±0.165 |
|  | Morita K | 2013 | Japan | GSE41874 | GPL7722 | 6 | 4 | 0.62±0.473 | | 1.30±0.237 |
|  | Su H | 2009 | China | GSE12717 | GPL7274 | 5 | 3 | 6.07±1.801 | | 8.16±0.040 |
|  | Kim J | 2012 | South Korea | GSE39678 | GPL15852 | 16 | 8 | 10.30±1.068 | | 10.72±0.292 |
|  | Villanueva A | 2016 | Spain | GSE74618 | GPL14613 | 218 | 10 | 1.32±0.115 | | 1.29±0.065 |
|  | Diaz G | 2013 | USA | GSE40744 | GPL14613 | 9 | 19 | 1.82±0.170 | | 1.65±0.166 |
|  | Burchard J | 2010 | USA | GSE22058 | GPL10457 | 96 | 96 | 1.50±0.342 | | 1.60±0.229 |
|  | TCGA | 2019 | USA | TCGA | TCGA | 375 | 50 | 12.30±1.11 | | 13.47±0.56 |
| hsa-miR-107 | Sato F | 2011 | Japan | GSE21362 | GPL10312 | 73 | 73 | 10.92±0.479 | | 10.52±0.249 |
|  | Xie Z | 2017 | China | GSE98269 | GPL20712 | 3 | 3 | 10.46±0.346 | | 10.36±0.281 |
|  | Morita K | 2013 | Japan | GSE41874 | GPL7722 | 6 | 4 | 1.21±0.356 | | 1.01±0.225 |
|  | Su H | 2009 | China | GSE12717 | GPL7274 | 5 | 3 | 13.48±0.734 | | 13.96±0.396 |
|  | Kim J | 2012 | South Korea | GSE39678 | GPL15852 | 16 | 8 | 12.06±0.464 | | 12.19±0.255 |
|  | Villanueva A | 2016 | Spain | GSE74618 | GPL14613 | 218 | 10 | 4.38±0.156 | | 4.29±0.072 |
|  | Diaz G | 2013 | USA | GSE40744 | GPL14613 | 9 | 19 | 5.33±0.144 | | 5.27±0.095 |
|  | Li W | 2008 | China | GSE10694 | GPL6542 | 78 | 88 | 14.60±0.586 | | 13.95±0.738 |
|  | Burchard J | 2010 | USA | GSE22058 | GPL10457 | 96 | 96 | 1.16±0.239 | | 1.00±0.105 |
|  | TCGA | 2019 | USA | TCGA | TCGA | 375 | 50 | 9.29±0.746 | | 8.78±0.366 |
| hsa-miR-17-5p | Sato F | 2011 | Japan | GSE21362 | GPL10312 | 73 | 73 | 9.80±0.944 | | 9.35±0.751 |
|  | Xie Z | 2017 | China | GSE98269 | GPL20712 | 3 | 3 | 9.42±0.954 | | 9.77±0.222 |
|  | Morita K | 2013 | Japan | GSE41874 | GPL7722 | 6 | 4 | 1.46±0.699 | | 0.71±0.223 |
|  | Su H | 2009 | China | GSE12717 | GPL7274 | 5 | 3 | 13.30±1.142 | | 12.71±0.215 |
|  | Kim J | 2012 | South Korea | GSE39678 | GPL15852 | 16 | 8 | 11.05±0.684 | | 10.41±0.201 |
|  | Villanueva A | 2016 | Spain | GSE74618 | GPL14613 | 218 | 10 | 4.76±0.537 | | 4.93±0.399 |
|  | Diaz G | 2013 | USA | GSE40744 | GPL14613 | 9 | 19 | 6.64±0.358 | | 6.85±0.265 |
|  | Li W | 2008 | China | GSE10694 | GPL6542 | 78 | 88 | 12.85±0.930 | | 12.32±0.709 |
|  | Burchard J | 2010 | USA | GSE22058 | GPL10457 | 96 | 96 | 0.69±0.268 | | 0.54±0.106 |
|  | TCGA | 2019 | USA | TCGA | TCGA | 375 | 50 | 12.41±0.955 | | 11.56±0.583 |
| hsa-miR-125a-5p | Sato F | 2011 | Japan | GSE21362 | GPL10312 | 73 | 73 | 8.18±1.119 | | 9.06±0.535 |
|  | Xie Z | 2017 | China | GSE98269 | GPL20712 | 3 | 3 | 8.32±1.720 | | 7.70±0.091 |
|  | Morita K | 2013 | Japan | GSE41874 | GPL7722 | 6 | 4 | 0.91±0.141 | | 1.19±0.134 |
|  | Su H | 2009 | China | GSE12717 | GPL7274 | 5 | 3 | 9.96±1.771 | | 11.40±0.414 |
|  | Kim J | 2012 | South Korea | GSE39678 | GPL15852 | 16 | 8 | 8.41±0.803 | | 9.84±0.588 |
|  | Villanueva A | 2016 | Spain | GSE74618 | GPL14613 | 218 | 10 | 3.63±0.453 | | 3.93±0.109 |
|  | Diaz G | 2013 | USA | GSE40744 | GPL14613 | 9 | 19 | 4.72±0.771 | | 5.64±0.479 |
|  | Li W | 2008 | China | GSE10694 | GPL6542 | 78 | 88 | 12.61±1.044 | | 12.77±0.973 |
|  | Burchard J | 2010 | USA | GSE22058 | GPL10457 | 96 | 96 | 0.82±0.247 | | 1.09±0.121 |
|  | TCGA | 2019 | USA | TCGA | TCGA | 375 | 50 | 10.24±1.073 | | 10.90±0.352 |
| hsa-miR-22-3p | Sato F | 2011 | Japan | GSE21362 | GPL10312 | 73 | 73 | 12.12±0.609 | | 12.46±0.322 |
|  | Xie Z | 2017 | China | GSE98269 | GPL20712 | 3 | 3 | 10.09±0.610 | | 11.16±0.250 |
|  | Morita K | 2013 | Japan | GSE41874 | GPL7722 | 6 | 4 | 1.13±0.683 | | 1.60±0.492 |
|  | Su H | 2009 | China | GSE12717 | GPL7274 | 5 | 3 | 12.69±1.039 | | 13.34±0.211 |
|  | Kim J | 2012 | South Korea | GSE39678 | GPL15852 | 16 | 8 | 12.21±0.747 | | 11.95±0.361 |
|  | Villanueva A | 2016 | Spain | GSE74618 | GPL14613 | 218 | 10 | 4.50±0.380 | | 4.60±0.261 |
|  | Diaz G | 2013 | USA | GSE40744 | GPL14613 | 9 | 19 | 5.65±0.373 | | 5.73±0.258 |
|  | Li W | 2008 | China | GSE10694 | GPL6542 | 78 | 88 | 13.02±1.007 | | 13.47±0.951 |
|  | Burchard J | 2010 | USA | GSE22058 | GPL10457 | 96 | 96 | 1.76±0.293 | | 1.94±0.118 |
|  | TCGA | 2019 | USA | TCGA | TCGA | 375 | 50 | 19.15±0.854 | | 19.54±0.464 |
