# Supplementary figures and images for: Identify and Validate the Transcriptomic, Functional Network, and Predictive Validity of FBXL19-AS1 in Hepatocellular Carcinoma
Source: Front Oncol. 2020 Dec 3;10:609601. doi: 10.3389/fonc.2020.609601 (PMC7744744; doi:10.3389/fonc.2020.609601)

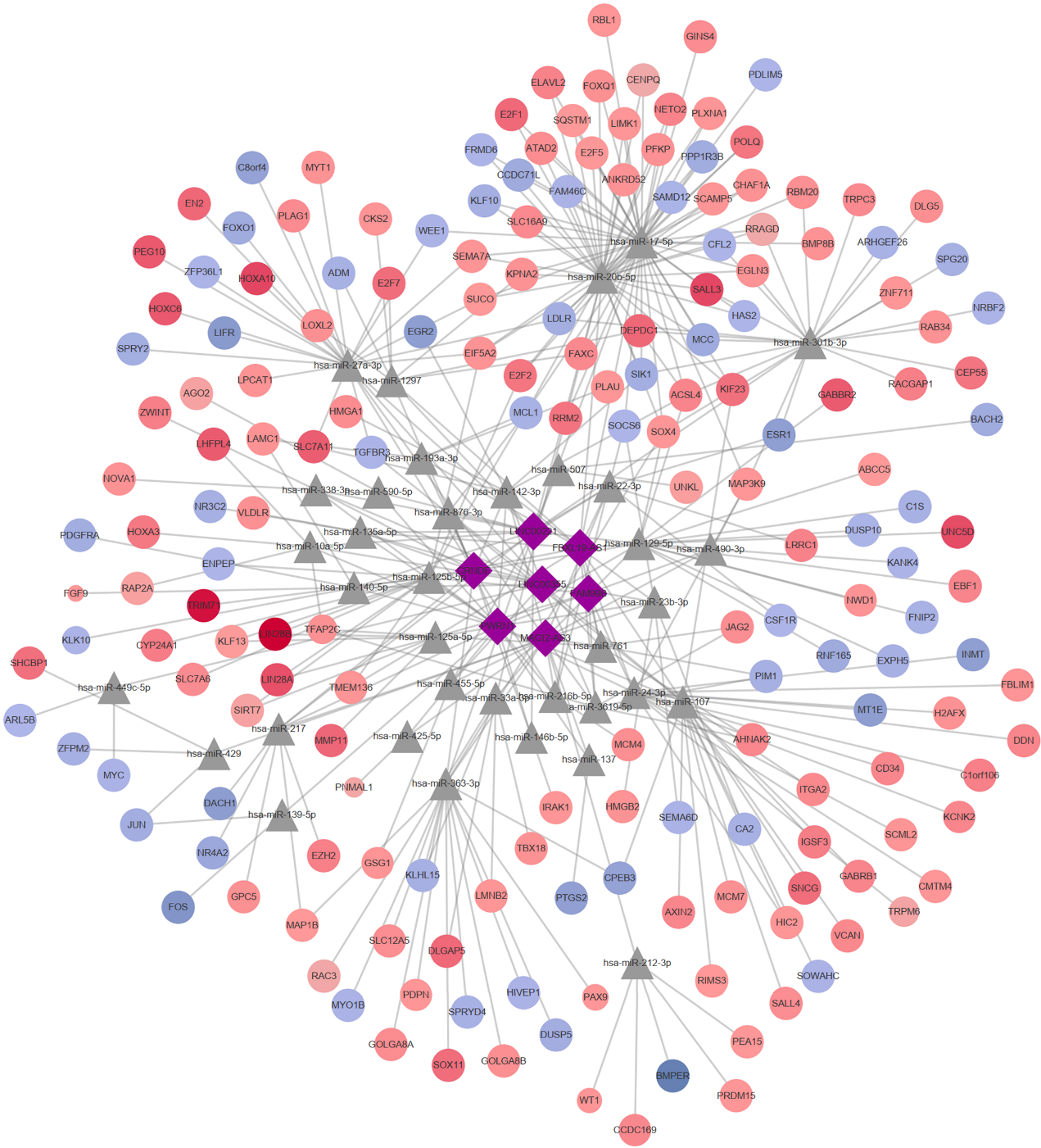


**Figure S1** CeRNA network of 7 lncRNAs.

Supplement: Supplementary file 1 [file DataSheet_1.zip › Supplementary material/Figure S1.docx]

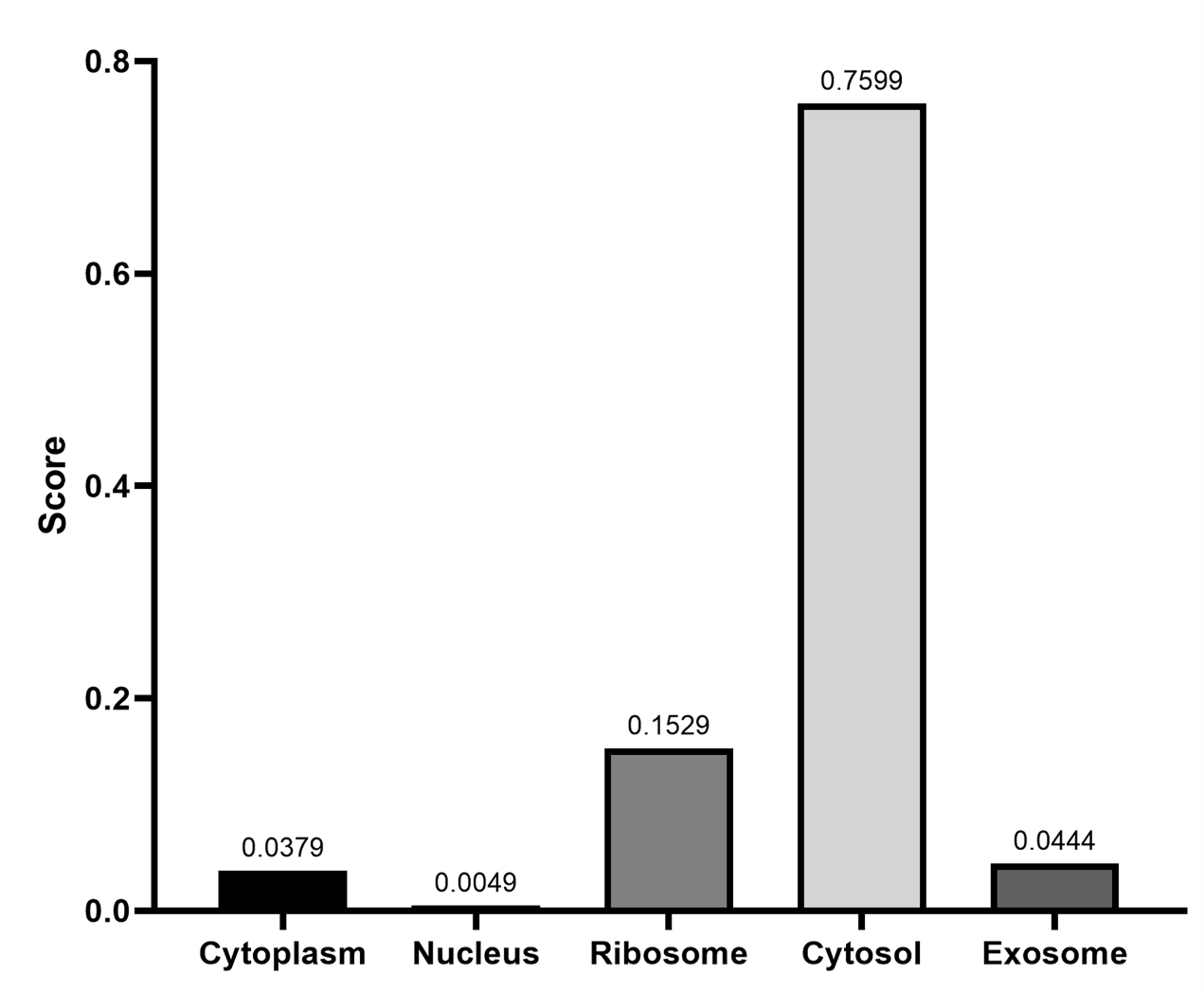


**Figure S2** Localization of FBXL19-AS in cells.

Supplement: Supplementary file 1 [file DataSheet_1.zip › Supplementary material/Figure S2.docx]

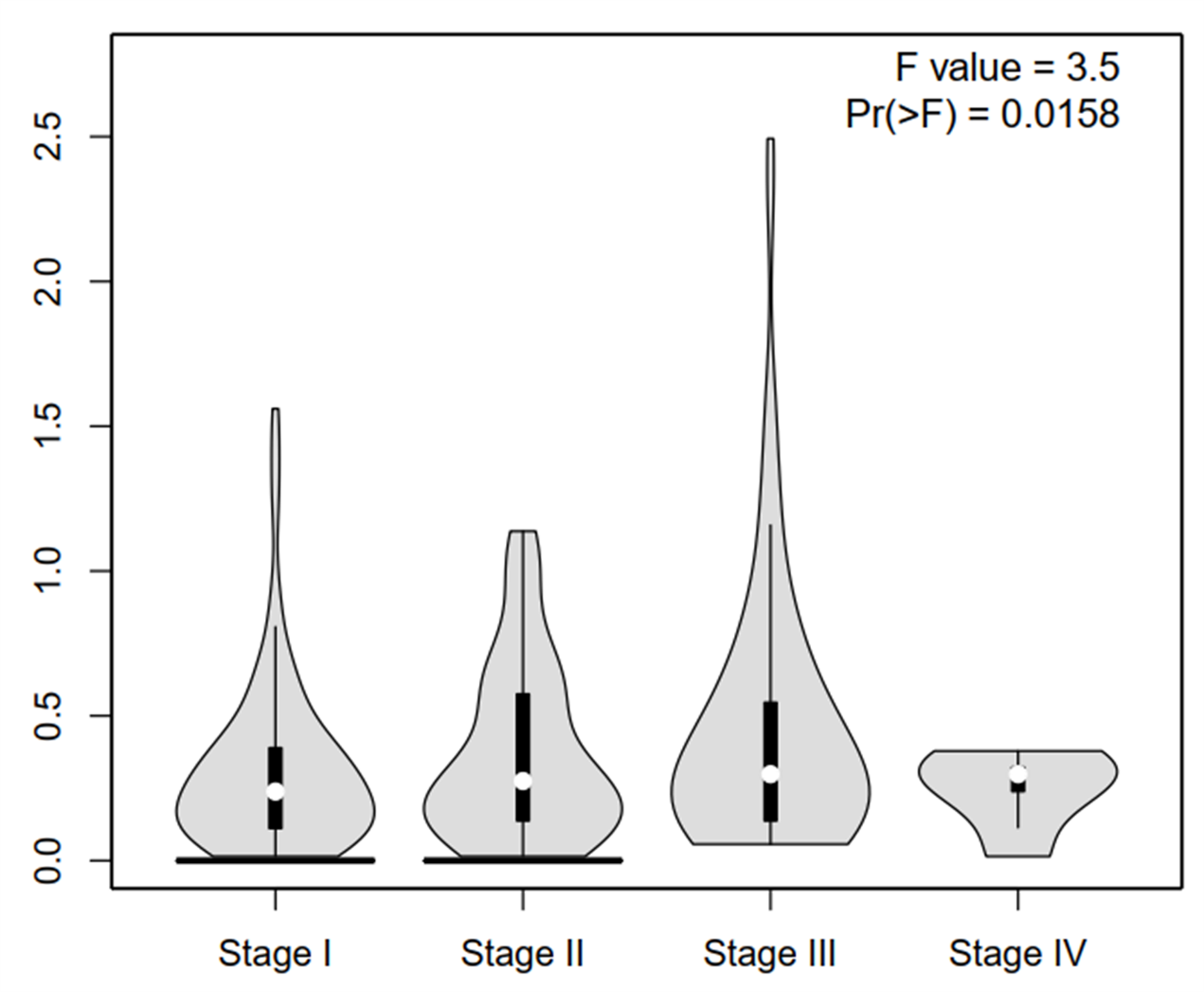


**Figure S3** Violin diagram of the relationship between FBXL19-AS1 and clinical stage.

Supplement: Supplementary file 1 [file DataSheet_1.zip › Supplementary material/Figure S3.docx]
